# Real-time 3D echocardiographic transilluminated imaging combined with artificially intelligent left atrial appendage measurement for atrial fibrillation interventional procedures

**DOI:** 10.3389/fphys.2022.1043551

**Published:** 2022-11-09

**Authors:** Aijiao Sun, Sihua Ren, Yangjie Xiao, Yixin Chen, Nan Wang, Chendi Li, Xueying Tan, Yilong Pan, Feifei Sun, Weidong Ren

**Affiliations:** ^1^ Department of Ultrasound, Shengjing Hospital of China Medical University, Shenyang, China; ^2^ Department of Radiology, The First Affiliated Hospital of China Medical University, Shenyang, China; ^3^ Department of Cardiology, Shengjing Hospital of China Medical University, Shenyang, China

**Keywords:** 3D Auto LAA, artificial intelligence-AI, left atrial appendage occlusion, real-time three-dimensional, transesophageal echocardiography, TrueVue Glass

## Abstract

**Aims:** This study investigated the feasibility and accuracy of real-time three-dimensional (3D) echocardiographic transilluminated imaging (TrueVue Glass) in left atrial appendage (LAA) anatomical morphology and artificial intelligence (AI)-assisted 3D automated LAA measurement (3D Auto LAA) software in the preoperative evaluation of LAA occlusion (LAAO) in patients with atrial fibrillation (AF).

**Method and results:** Thirty-seven patients with AF were selected. Two-dimensional (2D) and real-time 3D transesophageal echocardiography (RT3D-TEE) were performed preoperatively, using conventional 3D, the new 3D TrueVue Glass mode, and cardiac computed tomography angiography (CCTA) to assess and type the morphology of LAA. Physiological parameters were measured using traditional 2D and 3D manual (3D Manual LAA), 3D Auto LAA, and CCTA. TrueVue Glass for LAA outer contour display was compared with CCTA. Comparisons were based on correlation and consistency in measuring the maximum diameter (LZ max), minimum diameter (LZ min), area (LZ area), and circumference (LZ cir) of LAA landing zone (LZ). Times and variabilities were compared. The concordance rate for external shape of LAA was 97.14% between TrueVue Glass and CCTA. 3D Auto LAA and 3D Manual LAA have a stronger correlation and higher consistency in all parameters. 3D Auto LAA showed higher intra- and interobserver reproducibility and allowed quicker analysis (*p* < 0.05). LAAO was performed in 35 patients (94.59%), and none of which had serious adverse events.

**Conclusion:** TrueVue Glass is the first non-invasive and radiation-free visualization of the overall external contour of LAA and its adjacent structures. 3D Auto LAA simplifies the measurement, making the preoperative assessment more efficient and convenient while ensuring the accuracy and reproducibility. A combination of the two is feasible for accurate and rapid assessment of LAA anatomy and physiology in AF patients and has practical application in LAAO.

## 1 Introduction

Left atrial appendage occlusion (LAAO) has gained widespread acceptance and use as a new tool for stroke prevention in patients with atrial fibrillation (AF) (Holmes et al., 2019; Turagam et al., 2020). The 2020 European Society of Cardiology and European Association for Cardiothoracic Surgery guidelines for the diagnosis and management of AF give LAAO a class II b recommendation and a level of evidence of B (Hindricks et al., 2021). The anatomical structure of the LAA varies significantly among individuals, and thus the successful performance of LAAO depends on the accurate preoperative assessment of the morphology, size, and physiological status of the LAA (Korsholm et al., 2020). Currently, transesophageal echocardiography (TEE) and cardiac computed tomography angiography (CCTA) are the main methods for preoperative evaluation of the LAA. The main advantages of conventional two-dimensional (2D) and real-time three-dimensional (RT3D) TEE are that the spatial resolutions are significantly higher than that of transthoracic echocardiography, enabling TEE to clearly and dynamically display the internal structure of the LAA, identify the thrombus and comb muscles, and provide more accurate measurements. However, the assessment of the overall morphology can only be made based on lateral-view LAA cross-sectional images. In contrast, CCTA often must be combined with contrast-enhanced imaging; its main application value is in displaying the external contour of the LAA (Glikson et al., 2020; Korsholm et al., 2020). However, 3D image reconstruction is required to obtain LAA shape images, and LAA morphology changes cannot be shown with the cardiac cycle in real time, making this method time-consuming, laborious, and requiring high human interference. Previously, some researchers considered 2D-TEE as the gold standard for the preoperative measurement of relevant morphological parameters in LAAO, but with the rapid development of three-dimensional ultrasound, an increasing number of studies have shown that the results of RT3D-TEE are more accurate and reliable and correlate better with the implanted occluder, making it more suitable as a key reference for the selection of occluder models for LAAO patients (Morcos et al., 2018; Streb et al., 2019). However, in addition to its inability to directly display the overall external contour of the LAA, conventional RT3D-TEE still has several limitations such as cumbersome and time-consuming operation procedures when measuring the anatomical and physiological parameters of the LAA (Morais et al., 2022). Recently introduced advanced 3D echocardiographic imaging and measurement technologies, referred to as TrueVue Glass and 3D automated LAA (3D Auto LAA) measurement, are expected to solve the aforementioned problems. TrueVue Glass is a new 3D rendering mode that can intelligently render the contours of the heart chambers and vascular chambers containing blood flow and the heart valve structure, automatically shield substantial structures around the heart, and provide a new perspective for the ultrasonic diagnosis and evaluation of heart diseases (Karagodin et al., 2020). In contrast, 3D Auto LAA is an artificial intelligence (AI)-assisted automatic measurement technology that can automatically identify the ostium of the LAA after cutting and measure important parameters related to surgery such as the maximum diameter, minimum diameter, area, and circumference. To the best of our knowledge, their combined application in LAAO has not been systematically explored. In this study, for the first time, 3D Auto LAA was performed in association with TrueVue Glass for the examination and evaluation of AF patients undergoing LAAO and compared with other commonly used imaging techniques to explore the clinical value and technical advantages of these new methods.

## 2 Materials and methods

### 2.1 Study population

Thirty-seven patients with AF who were proposed to undergo LAAO from July 2020 to May 2022 at the Cardiology Center of China Medical University were included in this study, and all patients underwent 2D-TEE, RT3D-TEE, and CCTA preoperatively. The inclusion criteria are as follows: AF thrombotic risk score (CHA_2_DS_2_-VASc score) ≥ 2, while meeting one of the following conditions unsuitable for or unwilling to be subjected to long-term standardized anticoagulation; stroke or embolism occurring based on long-term standardized anticoagulation; bleeding risk score (HAS-BLED score) ≥ 3; and need for combined antiplatelet drug therapy. The exclusion criteria are 1) contraindication to TEE; 2) suspicious or definite thrombus in the LAA on preoperative TEE or CCTA; 3) presence of severe cardiac structural abnormalities or valvular disease requiring surgical treatment; 4) New York Heart Association cardiac function class IV; and 5) presence of recent active bleeding. This study was approved by our Institutional Ethics Committee and was conducted in accordance with the ethical principles for medical research involving human subjects established by the Declaration of Helsinki, protecting the privacy of all participants and the confidentiality of personal information.

### 2.2 Transesophageal echocardiography image acquisition and preoperative evaluation of left atrial appendage

In this study, a Philips CVx 3D ultrasound system (Philips Medical Systems, Andover, MA, United States) equipped with X7-2t and X8-2t transesophageal ultrasound probes was used for the 2D and 3D TEE examinations. All patients underwent TEE examinations and data acquisition after pharyngeal anesthesia, and 3–5 cardiac cycles were applied to store the images.

#### 2.2.1 2D-transesophageal echocardiography and RT3D-transesophageal echocardiography image acquisition

**FIGURE 1:**
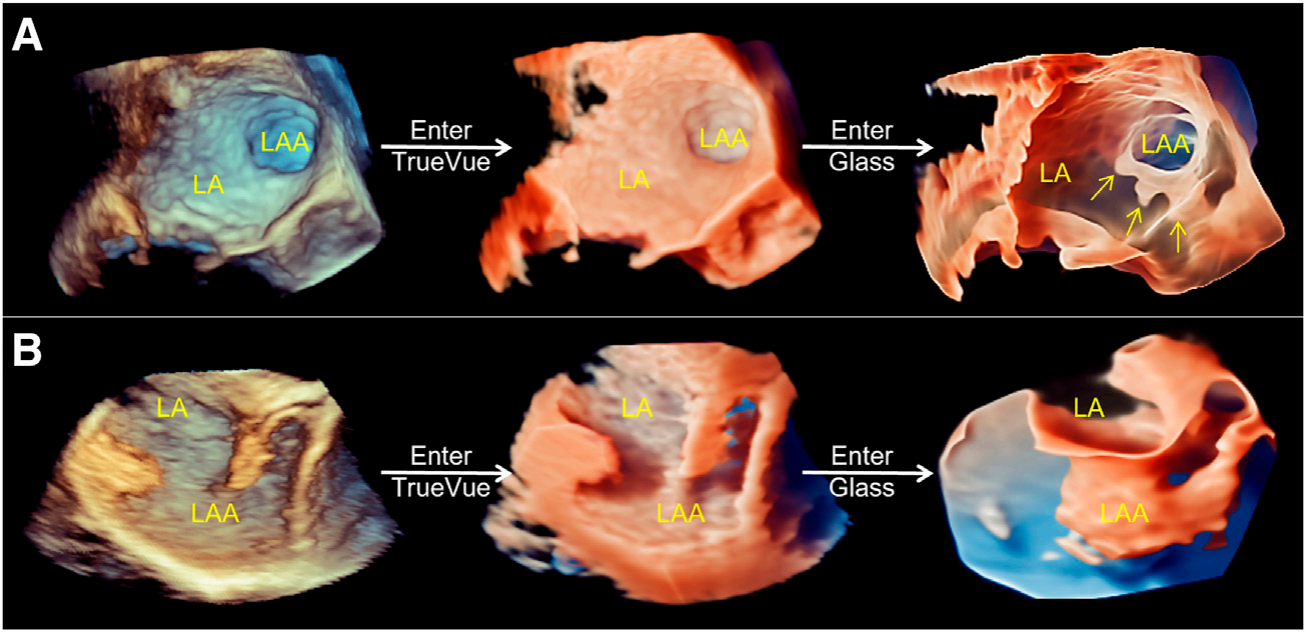
Schematic of process of the conversion of conventional real-time 3D transesophageal echocardiography to TrueVue Glass. **(A)**. From left to right, conventional 3D, TrueVue and TrueVue Glass imaging modes of the LAA at the entrance of the left atrium are shown. The TrueVue Glass (right) shows the three lobular structures at the blind end of the LAA (arrows) **(B)**. From left to right, conventional 3D, TrueVue and TrueVue Glass imaging of the LAA in lateral view are shown. 3D, three-dimensional; LA, left atrium; LAA, left atrium appendage.

2D-TEE was used to observe the morphology of the LAA, particularly the internal commissural muscle and blind end lobes in multiple angles and views, and to clarify the presence of thrombus and its relationship with surrounding adjacent structures. And 2D data acquisition was performed at 0°, 45°, 90°, and 135°. The 2D image depth, gain, and other parameters are adjusted to produce the best display output from the LAA display, and 3D Zoom mode is selected for 3D data acquisition of the complete structure of the LAA. The acquired LAA 3D data set is entered into TrueVue Glass mode *via* the instrument touch panel (first “TrueVue” is clicked upon, and then “Glass” is clicked upon to enter TrueVue Glass mode), and parameters, such as transparency, smoothness, contrast, and gain, are adjusted appropriately. The external contours of the LAA are clearly displayed using a suitable image cutting method (cutting tools as before (Sun et al., 2022)) (Figure 1).

#### 2.2.2 Preoperative evaluation of left atrial appendage occlusion related parameters for 2D-Transesophageal echocardiography and RT3D-Transesophageal echocardiography

**FIGURE 2:**
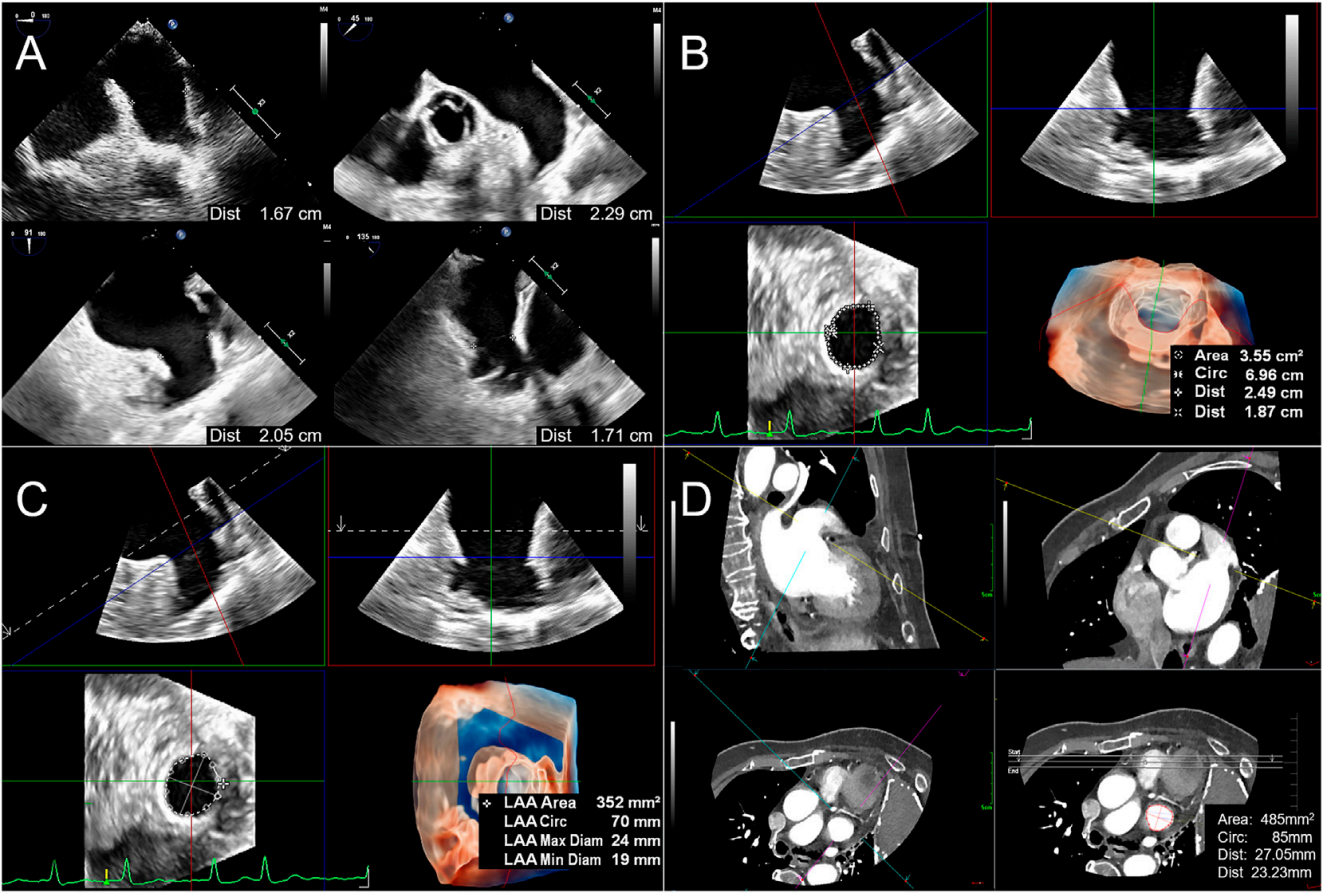
Flow chart of LAA parameter measurements before percutaneous left atrial appendage occlusion. **(A)**. For 2D TEE method, the LAA LZ diameters (LZ is defined as the point from the left circumflex artery to 1–2 cm below the contralateral left superior pulmonary vein crest) are measured at 0°, 45°, 90°, and 135 at end-systole, respectively. **(B)**. When applying 3D Manual LAA method, enter MultiVue mode on the basis of LAA 3D Zoom image, and at the end of systole, adjust the LZ positioning line (blue line) in the LAA sagittal plane (upper left), coronal plane (upper right), obtain the LAA LZ cross-section (lower left), and manually measure the LAA LZ maximum diameter (the first Dist value obtained), minimum diameter (the second Dist value), area and circumference (Circ). The lower right figure shows 3D downward view of the LAA LZ and the measurement results. **(C)**. After positioning the LAA LZ in the same way as 3D Manual LAA, the above parameters are automatically obtained by clicking “3D Auto LAA” (lower right). **(D)**. When using CCTA, measurement method is similar to 3D Manual LAA by adjusting the LZ positioning lines (yellow lines) in the sagittal plane (upper left) and coronal plane (upper right) of the LAA, obtaining the LAA LZ cross-section (lower left) and then manually measuring the above parameters in sequence (lower right). 3D Auto LAA, three-dimensional automated left atrial appendage; 3D Manual LAA, three-dimensional manual left atrial appendage; CCTA, cardiac computed tomographic angiography; LAA, left atrium appendage; LZ, landing zone.

The inner diameter of the LAA landing zone (LZ) was measured at 0°, 45°, 90°, and 135° at the end of the left ventricular systole (starting from the left circumflex coronary artery to 1–2 cm below the contralateral left superior pulmonary vein crest). The maximum value of the inner diameter in the four angles was labeled LZ max, and the minimum value was labeled LZ min of 2D-TEE (Figure 2A). LAA anatomical parameter data measure of RT3D-TEE was performed online, including in the traditional 3D manual measurement method (3D Manual LAA) and intelligent measurement method (3D Auto LAA). 3D Manual LAA measurement method: The appropriate LAA 3D Zoom image was selected, and MultiVue mode was entered. The end-systolic phase of left ventricle was selected, and the blue line (LAA LZ cross-sectional positioning line) in the sagittal and coronal planes of LAA, thus enabling the LAA LZ ostium to be obtained. In this plane, the LAA LZ maximum diameter (LZ max), minimum diameter (LZ min), area (LZ area), and circumference (LZ cir) are manually measured (Figure 2B). 3D Auto LAA measurement method: 3D Auto LAA can automatically identify the endocardium, after adjusting the LAA LZ in MultiVue mode as shown in 3D Manual LAA, the above anatomical and physiological parameters of the LAA LZ can be automatically obtained with a single click on “3D Auto LAA” (Figure 2C). Manual adjustment to identify unsatisfactory loci when needed is allowed.

### 2.3 Cardiac computed tomography angiography image acquisition and measurements

An IQon-Spectral CT instrument from Philips was used for cardiac enhancement imaging and to display the external morphology of the LAA *via* 3D reconstruction. In the LAA measurement method, which is similar to conventional RT3D-TEE, the LAA LZ is manually adjusted from three mutually perpendicular planes, and the LZ max, LZ min, LZ area, and LZ cir parameters are measured separately (Figure 2D).

### 2.4 Comparison of time

The time taken to measure the LAA by each of the aforementioned methods (specifically, the time period from loading and displaying data on the workstation to extraction for all clinical indicators) is recorded.

### 2.5 left atrial appendage morphological determination

The international anatomical morphology classification of LAA into four types is as follows (Wang et al., 2010): 1) chicken-wing type: LAA has the main leaf with a clear bend at its proximal or middle part and may have secondary lobes; 2) windsock type: LAA has no clear bend, has a sufficiently long main leaf, and can vary according to the position and number of secondary and tertiary leaves issued; 3) cactus type: LAA has no clear bend and is characterized by a dominant primary leaf, with secondary leaves extending upward or downward, and 2–3 secondary leaves at the tip of one primary leaf; and 4) cauliflower type: LAA without obvious curvature and with limited overall length, lack of primary leaves, more complex internal structure, and irregular shape of LAA mouth.

### 2.6 Procedural strategy for left atrial appendage occlusion

The patient is delivered with a sheath and guidewire *via* the femoral vein under general anesthesia, followed by penetration of the guidewire through the interatrial septum under X-ray fluoroscopy, TEE, or intracardiac echocardiography guidance and delivery to the LAA for contrast observation. The choice of the occluder model is recommended to be 4–6 mm larger than the maximum LZ diameter measured by intraoperative fluoroscopy or TEE. After the initial implantation is completed, it is judged that the occluder is appropriately positioned: there is no residual shunt or shunt bundle width <5 mm around it, pull test is stable, compression ratio is appropriate, and release principle is satisfied before it is fully deployed (Vainrib et al., 2018), while observations are made based on whether adverse events such as pericardial effusion and compression of surrounding tissue structures have occurred. The procedure is ended after it is determined that there are no abnormalities.

### 2.7 Reproducibility verification and follow-up

To determine the reproducibility of the different methods of preoperatively measuring the parameters of LAA, measurements were repeated one month later in 10 randomly selected patients by the same observer and by a different observer. During the repeat analysis, the observers were unaware of all previous measurements and clinical details. TEE follow-up was applied approximately 45 days, 3 months and 6 months after surgery to observe the morphology and position of the occluder and the occurrence of adverse events such as residual shunts and device-related thrombosis around the blocker.

### 2.8 Statistics

Statistical analysis was performed using SPSS Statistics version 26.0 (IBM Inc., Armonk, New York). The normal distribution of continuous quantitative variables was assessed *via* the Kolmogorov–Smirnov test. Continuous variables are presented as mean ± standard deviation or median (first interquartile range [IQR], third interquartile range) for skewed variables, and categorical variables are presented as counts and percentages. A comparison of the results obtained by the different methods of measuring the preoperative anatomical parameters of LAA was performed *via* paired-sample Wilcoxon signed-rank test and using the Pearson correlation and intraclass correlation coefficient (ICC). The ICC was used to determine the intra- and interobserver variability, and 95% confidence intervals (CI) were calculated. It is generally accepted that an ICC below 0.4 indicates poor agreement (reliability) and above 0.75 indicates high agreement (reliability). One-way analysis of variance was then used to compare the differences in the times taken to measure LAA using the different measurement methods, where *p* < 0.05 was considered statistically significant.

## 3 Results

Patient demographics: Of the 37 AF patients, 35 (94.59%) had successful final LAAO surgery, whereas two had their procedures terminated because of intra-LAA thrombosis on preoperative TEE. The basic patient characteristics are presented in Table 1.

**TABLE 1:** Baseline clinical characteristics of study population (*n* = 35).

Parameter	Value
Men, n (%)	19 (54.29%)
Age, years	61.20 ± 9.98
Medical history	
Paroxysmal AF, n (%)	23 (65.71%)
Nonparoxysmal AF, n (%)	12 (34.29%)
Hypertension, n (%)	25 (71.43%)
Diabetes mellitus, n (%)	12 (34.29%)
Coronary Heart Disease, n (%)	5 (14.29%)
Prior stroke/TIA, n (%)	4 (11.43%)
CHA_2_DS_2_-VASc score	3.20 ± 1.32
HAS-BLED score	2.17 ± 0.92

All data are presented as number (%) or mean ± SD. AF, atrial fibrillation; TIA, transient ischemic attack. CHA_2_DS_2_-VASc score is a scoring for stroke risk assessment in patients with atrial fibrillation (C: 1 point for Congestive heart failure; H: 1 point for Hypertension; A2: 2 points for Age ≥75 years; D: 1 point for Diabetes; S2: 2 points for thromboembolism, Stroke, or transient ischemic attack; V: 1 point for Vascular disease; A: 1 point for Age of 65–74 years; S: sex category, 1 point for female). HAS-BLED score is a scoring scale for bleeding risk in patients with atrial fibrillation (For the presence of Hypertension, Abnormal renal/liver function, Stroke, Bleeding history, Labile INRs, Elderly (>65 years), or Drugs/alcohol, 1 point was recorded).

### 3.1 Comparison of left atrial appendage morphology by TrueVue Glass and cardiac computed tomography angiography

**FIGURE 3:**
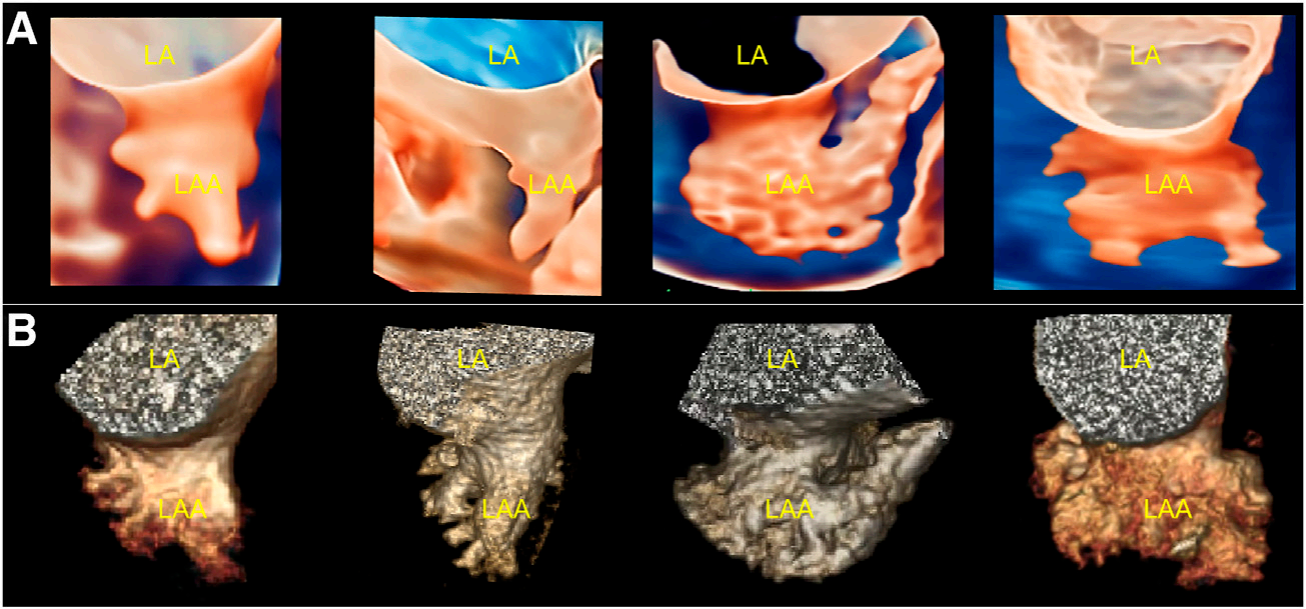
Comparison of TrueVue Glass and CCTA for displaying different morphologies of LAA. **(A)**. From left to right, TrueVue Glass shows cactus, windsock, chicken-wing, and cauliflower type of LAA **(B)**. From left to right, CCTA display of the above morphological types of LAA are shown. CCTA, cardiac computed tomographic angiography; LA, left atrium; LAA, left atrium appendage.

From all patients, stereo morphological information of the LAA was obtained using TrueVue Glass and CCTA images. Additionally, systolic and diastolic changes in the LAA with cardiac pulsation can be observed in real time using TrueVue Glass (Supplementary Movie S1). TrueVue Glass revealed 11/35 (31.43%) cases of cactus type, 10/35 (28.57%) cases of windsock type, 9/35 (25.71%) cases of chicken-wing type, and 5/35 (14.29%) cases of cauliflower type. For comparison, CCTA revealed 12/35 (34.29%) cases of cactus type, 9/35 (25.71%) cases of windsock type, 9/35 (25.71%) cases of chicken-wing type, and 5/35 (14.29%) cases of cauliflower type. One case was determined by TrueVue Glass to be windsock type and CCTA to be cactus type; the compliance rate was 34/35 (97.14%) (Figure 3). In addition, some patients with AF showed spontaneous echo contrast because of the enlarged left atrium and LAA; the flowing cloudy stereomorphic features could be observed using TrueVue Glass (Supplementary Movie S2).

### 3.2 Comparison of 3D auto LAA with 2D-Transesophageal echocardiography, 3D manual left atrial appendage, and cardiac computed tomography angiography

3D Auto LAA was feasible for all patients, although 19 patients (54.29%) required fine-tuning of the recognition position by hand at the time of analysis.

The variability, correlation, and agreement between 3D Auto LAA and other measurement methods are presented in Table 2. There were no significant differences in LZ max and LZ min between 3D Auto LAA and 2D-TEE (*p* > 0.05), whereas there were significant differences in LZ max and LZ min between 3D Auto LAA and 3D Manual LAA (*p* < 0.05). In contrast, there were no significant differences in LZ area and LZ cir between 3D Auto LAA and 3D Manual LAA (*p* > 0.05). There were significant differences in LZ max, LZ min, LZ area, and LZ cir between 3D Auto LAA and CCTA (*p* < 0.05). 3D Auto LAA and 3D Manual LAA have a stronger correlation and higher consistency in all parameters, particularly for LZ area and LZ cir.

**TABLE 2:** Comparison of 2D-TEE, 3D Manual LAA, 3D Auto LAA and CCTA for preoperative measurements of parameters related to left atrial appendage occlusion.

	LZ max (mm)	LZ min (mm)	LZ area (mm^2^)	LZ cir (mm)
2D-TEE	21.35 (18.65, 22.88)	17.70 (14.75, 19.60)	NA	NA
3D Manual LAA	22.85 (19.83, 25.28)^a^	17.70 (15.03, 20.80)^a^	341.00 (206.25, 405.00)	67.05 (54.38, 74.05)
3D Auto LAA	21.50 (18.75, 24.00)	17.00 (13.50, 20.00)	332.50 (184.75, 375.00)	68.00 (56.00, 73.50)
CCTA	25.20 (21.03, 27.13)^a^	19.65 (17.78, 21.23)^a^	394.50 (302.75, 486.50)^a^	74.35 (65.20, 82.43)^a^
SCC (*p-*value)^b^	0.82 (<0.001)	0.73 (<0.01)	NA	NA
ICC (*p-*value)^b^	0.78 (<0.001)	0.66 (<0.01)	NA	NA
SCC (*p-*value)^c^	0.94 (<0.001)	0.95 (<0.001)	0.96 (<0.001)	0.96 (<0.001)
ICC (*p-*value)^c^	0.91 (<0.001)	0.89 (<0.001)	0.96 (<0.001)	0.96 (<0.001)
SCC (*p-*value)^d^	0.62 (0.02)	0.25 (0.40)	0.71 (0.01)	0.67 (0.01)
ICC (*p-*value)^d^	0.51 (0.01)	0.17 (0.20)	0.55 (<0.01)	0.55 (<0.01)

LZ max, LZ min, LZ area, and LZ cir are expressed as median (25th percentile, 75th percentile). 2D-TEE, two-dimensional transesophageal echocardiography; 3D Auto LAA, three-dimensional automated left atrial appendage; 3D Manual LAA, three-dimensional manual left atrial appendage; CCTA, cardiac computed tomographic angiography; ICC, intraclass correlation coefficient; LZ area, landing zone area; LZ cir, landing zone circumference; LZ max, landing zone maximum diameters; LZ min, landing zone minimum diameters; SCC, spearman correlation coefficient.

^a^ *p* < 0.05 vs. 3D Auto LAA in difference.

^b^ comparison results between 3D Auto LAA and 2D-TEE.

^c^ comparison results between 3D Auto LAA and 3D Manual LAA.

^d^ comparison results between 3D Auto LAA and CCTA.

### 3.3 Analysis time

**FIGURE 4:**
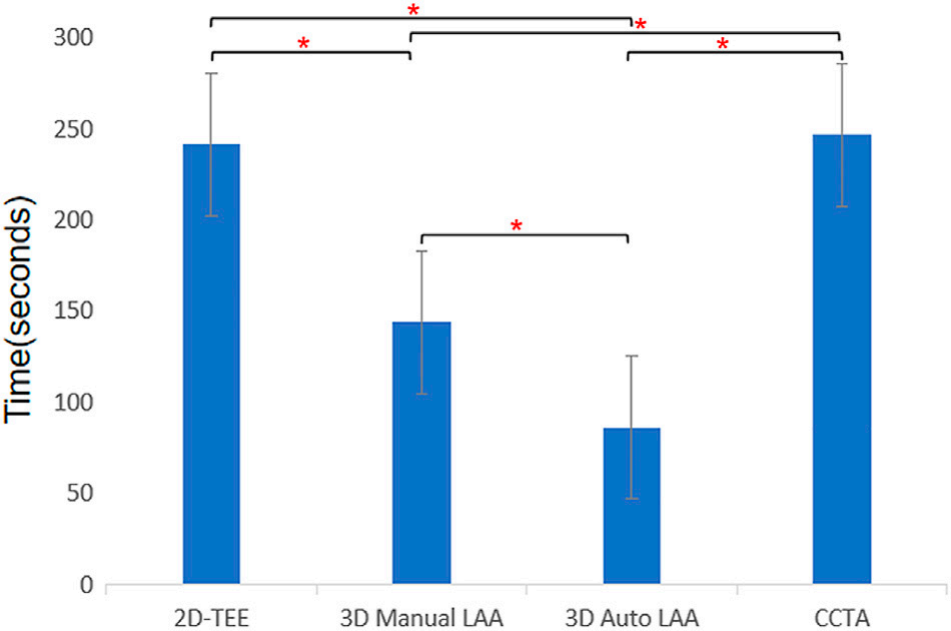
Times consumption of different methods for measuring morphological parameters of left atrium appendage. 2D-TEE, two-dimensional transesophageal echocardiography; 3D Auto LAA, three-dimensional automated left atrial appendage; 3D Manual LAA, three-dimensional manual left atrial appendage; CCTA, cardiac computed tomographic angiography; * *p-*value < 0.05.

Figure 4 shows the times taken to measure the LAA preoperative parameters for the different operating mode approaches that were evaluated. The time taken to measure LAA using 2D-TEE, 3D Manual LAA, 3D Auto LAA, and CCTA was 240.95 ± 0.20 s, 143.64 ± 0.31 s, 85.96 ± 0.42 s, and 246.33 ± 0.57 s, respectively. Except for 2D-TEE and CCTA, there were significant differences in operation time between the measurement modes (*p* < 0.05).

### 3.4 Reproducibility verification and follow-up

**FIGURE 5:**
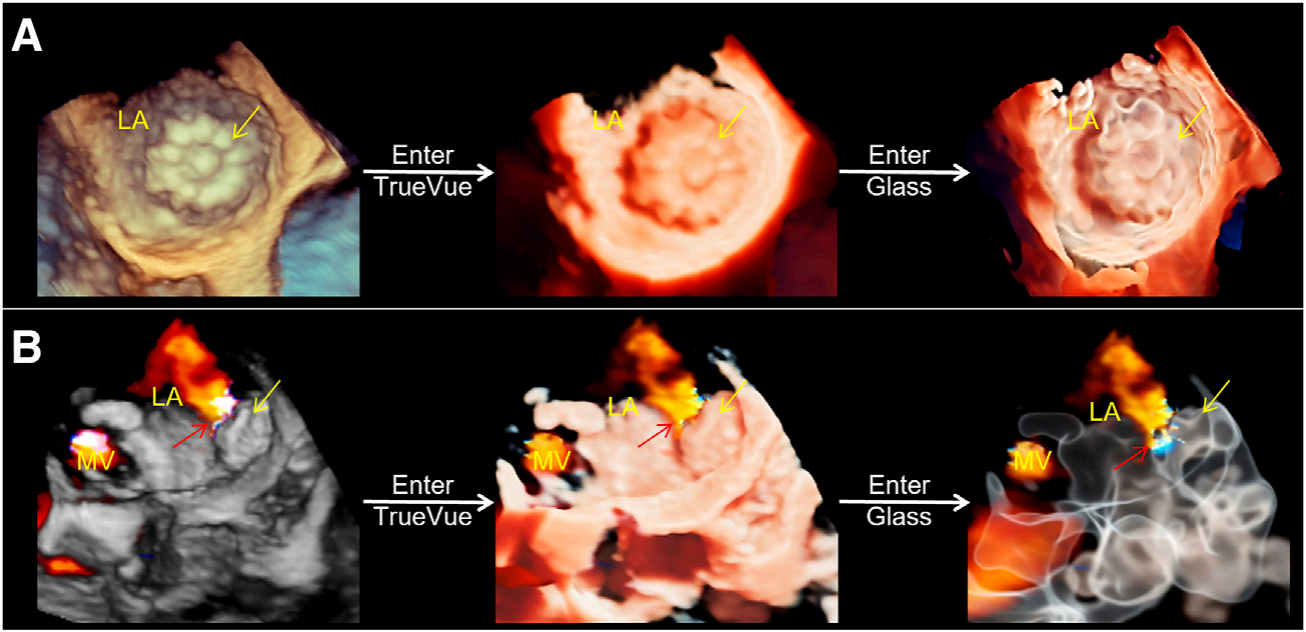
Three-dimensional transesophageal echocardiography plus color Doppler for outcome assessment after left atrial appendage occlusion. **(A)**. From left to right, conventional 3D, TrueVue and TrueVue Glass direct views of the left atrium appendage occluder (yellow arrows) are displayed. **(B)**. From left to right, conventional 3D, TrueVue and TrueVue Glass with color Doppler showing the residual shunt (red arrows) around the occluder (yellow arrows), among them, TrueVue Glass showing the origin and path of the shunt more entirely. LA, left atrium; MV, mitral valve.

Table 3 summarizes the intra- and interobserver variability of the different methods for preoperatively measuring the parameters of LAA. The results showed that 3D Auto LAA achieved higher interobserver and intraobserver reproducibility compared with those of 2D-TEE, 3D Manual LAA, and CCTA. At 45 days postoperatively, thirty-five patients were followed up with TEE (Figure 5A), and residual shunts around the occluder were found in six cases (17.14%), none of which exceeded 5 mm (considered to be ineffective shunts), and none of which had serious adverse events such as pericardial tamponade and device dislodgement. The application of conventional 2D, 3D-TEE, and TrueVue Glass all enable the diagnosis of residual shunts with no difference in diagnostic rates, but TrueVue Glass imaging allows the observation of the overall path of the shunt, including the overall spatial situation of the origin and travel of abnormal blood flow, through increased transparency (Figure 5B). At the 3-month postoperative follow-up, the residual shunts around the occluder disappeared in the six patients mentioned above. Thirty-four patients (97.14%) completed the 6-month postoperative follow-up and none of which had serious adverse events.

**TABLE 3:** Intra- and interobserver variability of 2D-TEE, 3D Manual LAA, 3D Auto LAA and CCTA.

	Intraobserver, ICC (95% CI)	Interobserver, ICC (95% CI)
2D-TEE		
LZ max	0.78 (0.36–0.94)	0.64 (0.03–0.90)
LZ min	0.67 (0.16–0.91)	0.15 (-0.36–0.66)
3D Manual LAA		
LZ max	0.72 (0.25–0.92)	0.64 (0.09–0.90)
LZ min	0.78 (0.35–0.94)	0.58 (-0.07–0.88)
LZ area	0.82 (0.42–0.95)	0.81 (0.44–0.95)
LZ cir	0.82 (0.42–0.95)	0.82 (0.47–0.95)
3D Auto LAA		
LZ max	0.81 (0.44–0.95)	0.72 (0.25–0.92)
LZ min	0.84 (0.51–0.96)	0.76 (0.33–0.93)
LZ area	0.87 (0.56–0.97)	0.86 (0.54–0.96)
LZ cir	0.87 (0.55–0.97)	0.83 (0.47–0.95)
CCTA		
LZ max	0.75 (-0.04–0.94)	0.50 (-0.19–0.85)
LZ min	0.50 (-0.14–0.85)	0.43 (-0.30–0.82)
LZ area	0.59 (0.04–0.88)	0.62 (-0.01–0.89)
LZ cir	0.59 (0.04–0.88)	0.49 (-0.21–0.85)

2D-TEE, two-dimensional transesophageal echocardiography; 3D Auto LAA, three-dimensional automated left atrial appendage; 3D Manual LAA, three-dimensional manual left atrial appendage; CCTA, cardiac computed tomographic angiography; ICC, intraclass correlation coefficient; LZ area, landing zone area; LZ cir, landing zone circumference; LZ max, landing zone maximum diameters; LZ min, landing zone minimum diameters.

## 4 Discussion

The main findings of this study include 1) TrueVue Glass is feasible for the stereoscopic display for the overall external contour of the LAA and adjacent structures, and has a high consistency with CCTA; 2) TrueVue Glass can also be used in the postoperative follow-up of LAAO patients to achieve a visual and integrated visualization of the residual shunt in and around the occluder; 3) AI-assisted automated LAA measurements are feasible, more efficient and reproducible than other previously used techniques. 3D Auto LAA results showed higher correlation with 3D Manual LAA method, which is generally considered to be more accurate, and there is no statistical difference between them in terms of important anatomical and physiological parameters of the LAA.

TrueVue Glass imaging is a new 3D cardiac ultrasound rendering mode that has been introduced in recent years (Karagodin et al., 2020; Sun et al., 2021). It automatically hides surrounding cardiac tissues through a one-click operation and presents the heart chambers and small and large vessel chambers containing blood flow in a crystal-clear visualization. It breaks through the limitations of conventional echocardiographic display, providing a new perspective not previously offered by 3D cardiac ultrasound, particularly for the overall external contour of the LAA, which is comparable to that of enhanced CT, and is a non-invasive, real-time dynamic imaging method.

The significant differences in LAA morphology pose several challenges for LAAO to proceed, especially the chicken-wing-type LAA, which is a particular challenge for LAAO (Freixa et al., 2013; Korsholm et al., 2018). Combined with recent studies (Hahn et al., 2022), the preoperative application of TrueVue Glass for LAAO can provide new information to support the assessment of the anatomical morphology of the LAA, i.e., the overall contour of the LAA and its adjacent structures from the outside. Karagodin et al. applied this technique to observe an LAA and confirmed that it could clearly show the LAA boundary contour and lobing, but the morphology of the LAA was displayed only from the lateral view, without further 3D display of the overall shape. This outcome was similar to the traditional 3D observation view and was not compared with CT results (Karagodin et al., 2020). In our study, the display rate of the overall contour morphology of the LAA by TrueVue Glass was 100%, and the results were basically consistent with the CCTA display results. Additionally, the spatial position relationship and hemodynamics of the surrounding structures, such as the left atrium, mitral valve, and aortic valve, could be displayed. To demonstrate, the anatomical features of the beating heart were displayed in a physiological state in real time (Supplementary Movie S1).

In the TrueVue Glass mode, a color doppler can be added, and after effective adjustment of transparency, the complete origin and path of the blood flow bundle can be observed through transparent tissue, thus locating more comprehensively and precisely any leaks around the LAAO postoperative occluder. Thus, the display of color blood flow is more comprehensive and clearer. In a past study, Tamborini et al. used TrueVue Glass in patients undergoing mitral valve repair and clearly observed the advantages of this method vs. conventional RT3D-TEE imaging in depicting the preoperative regurgitant orifice boundary and identifying the residual shunt after surgery, confirming the value of this technology in interventional procedures for various cardiac diseases (Tamborini et al., 2021). In our study, this advanced 3D cardiac ultrasound technology was systematically applied to the preoperative and postoperative real-time imaging evaluation of LAAO in patients with various forms of LAA, to observe whether the morphology and position of the occluder are normal and to qualify, locate, and quantify any residual leakages around the occluder, providing a basis for individualized treatment planning after surgery. Although the application of TrueVue Glass in this study did not differ from conventional RT3D-TEE in terms of the diagnostic rate of residual shunts, TrueVue Glass revealed a more realistic, comprehensive, and clearer overall path for residual shunts, improving diagnostic efficacy and communication with clinicians.

The advantages of TrueVue Glass over CCTA are three-fold. First, it is based on simple post-processing of conventional RT3D-TEE data and does not require additional examination of the patient. Second, it avoids damage to the patient caused by radiation or contrast allergy and avoids contraindications to CCTA imaging. Finally, it facilitates real-time dynamic imaging and provides timely diagnosis and evaluation of the patient at the bedside. This, with the gradual improvement of TrueVue Glass resolution and other parameters, is expected to replace the need for CT examination for LAAO surgery, to achieve a single ultrasound examination that completes the collection of information on various aspects of LAA anatomical morphology and physiological function and parameter measurement.

In our study, the LAA morphological classification of one patient was inconsistent with the CCTA results. The reason for this was inferred to be the poor quality of the original ultrasound image acquisition and the limitations of TrueVue Glass in resolving minute details, which resulted in the poor display of small LAA fractions located in the far field of the image. These inadequacies may have led to the difference in the identification of the overall morphological classification. Meanwhile, the LAA fractions of most patients were matched with the CT results commonly used in current clinical practice, and thus we considered the effect of the difference for this one patient to be minor.

To the best of our knowledge, this is the first study to explore the clinical application of 3D Auto LAA. This technique is a new three-dimensional fully automated LAA parameter measurement method based on TEE images, which can automatically track the endothelial border according to the shape of the LAA LZ of the patient and measure all important surgical reference indicators of the LAA LZ. If necessary, the examiner can make overall or local adjustments to the endothelial border to make the measurement results more accurate. The results of this study show that 3D Auto LAA reduces the number of manual tracing steps during traditional 3D measurements, resulting in a more convenient process, a significant reduction in analysis time (including when manual adjustments are required), and satisfactory reproducibility. Similar to other studies, the application of automated measurement techniques combined with manual fine-tuning allows for accurate measurements with higher repeatability (Queirós et al., 2018; Morais et al., 2022).

In this study, the measurements of the LZ max, LZ min, LZ area, and LZ cir of LAA by 3D Auto LAA correlated more strongly and agreed better with 3D Manual LAA than with 2D-TEE and CCTA, probably because both were usually measured based on the same raw 3D data. However, the differences between the two on LZ max and LZ min measurements of the LAA were significant. In contrast, there were no significant differences between the two on LZ area and LZ cir measurements, probably because of the diversity of LAA orifice morphology such as the oval, teardrop, and foot shape (Glikson et al., 2020). Moreover, the human eye is more prone to bias when measuring the maximum and minimum diameters, whereas it has little influence on the measurement after the LZ area and LZ cir are traced *via* manual or automatic methods. In addition, the parameters of LZ area and LZ cir may be more accurate for the selection of the occluder model compared to LZ max and LZ min. In a past study, Al-Kassou et al. (2017) showed that using the diameter derived from the LZ area and LZ cir as a reference indicator for the occluder model is more relevant to the final choice of the occluder model. Kong et al. also showed (Kong et al., 2020) that the diameter derived from the LAA circumference is more suitable as a reference indicator for the occluder model because it is more stable compared to other indicators.

In addition, although there were no significant differences between 3D Auto LAA and 2D-TEE in terms of the measured LZ max after multi-angle measurements in this study, numerically, the 3D Auto LAA measurements were still greater than 2D-TEE. This difference is probably because of the inherent display limitations of 2D-TEE, which sometimes does not take the maximum value from only four angles (Wunderlich et al., 2015; Song et al., 2016; Zhang et al., 2019). These limitations in 2D-TEE may lead to small device selection, resulting in excessive residual shunts and even dislodgment of the occluder, prolonging surgical time and negatively affecting the surgical outcome. The 3D Auto LAA avoids the above situation by measuring the LAA as a three-dimensional aspect, making the measurement results closer to the real anatomical parameters. X-ray is also observed and measured in a 2D plane and therefore faces limitations similar to those of 2D-TEE. Saw’s study showed that X-ray angiography consistently gave the lowest LAA diameters compared to CCTA and TEE and believed that X-ray angiographic measurements should have been omitted (Saw et al., 2016). It has been shown that the correlation between X-ray measurements and the final implanted occluder size of LAAO is the lowest, second to the 2D-TEE reference index, and the best correlation is with the 3D-TEE (Al-Kassou et al., 2017). In a recent expert consensus on LAAO released by EHRA and EAPCI in 2020, it was stated that CCTA measurements were the highest and the most accurate predictor of occluder size when comparing the results of the three imaging techniques (including TEE, CCTA and X-ray angiography) (Glikson et al., 2020). Therefore, a more appropriate radiological index-CCTA than X-ray angiography was chosen for reference in our study. Compared with TEE, which was the previously accepted gold standard for preoperative LAAO examination, CCTA has higher spatial resolution (Bai et al., 2017; Glikson et al., 2020) and in recent years has been suggested to become the new gold standard for the preoperative planning of LAAO (Korsholm et al., 2020). Meanwhile, researchers still believe that the measurement results of conventional 3D-TEE are more informative for the selection of LAAO occluder models (Morcos et al., 2018). Although CCTA also measures the LAA at the 3D level, the results of this study showed that the discrepancy and agreement between its measurements and 3D Auto LAA were poor compared to those of other methods, and the measurements were generally higher than those of TEE, which is consistent with the results of previous studies (Bai et al., 2017; Gilhofer and Saw, 2020; Glikson et al., 2020). This may be because of inherent differences in the imaging principles of the techniques or the fact that the application of CCTA requires the use of contrast agents, which inevitably infiltrate the local myocardium in the contrast-filled space, resulting in a large discrepancy in the measured values.

Therefore, combined with joint 2D and 3D-TEE to observe the presence of thrombus in the LAA from the inside, to observe the distribution and number of lobes of the commissural muscle at the blind end, and to measure the size and functional assessment of the LAA, we can achieve a comprehensive dynamic and accurate evaluation of the physiological structure and function of the LAA. This assessment is more conducive to designing an individualized occluder implantation plan in advance, anticipating possible risks that may be encountered during the implantation process and improving the efficiency and success rate of LAAO. Most of the patients in this study achieved complete closure of LAA, although six patients had residual shunts after surgery, but all of them were ineffective, which further proved the advantages of applying the combined technique.

### 4.1 Limitations

There are several limitations of our study. First, the sample size of this study was small and the follow-up period is relatively short, and although it initially confirmed the superior role of TrueVue Glass and 3D Auto LAA in LAAO, further validation using a larger sample size and longer follow-up is necessary. Second, although the visualization of the LAA by TEE has basically fulfilled clinical requirements, 3D Auto LAA is still dependent on the quality of the original 2D ultrasound image. When the image quality is poor, or when there are obvious spontaneous echo contrasts within the LAA, it may interfere with the accurate identification of the endothelium by 3D Auto LAA. This interference may result in small measurement values, which require minor manual adjustments of the tracking position by the sonographer. Third, at present, TrueVue Glass and 3D Auto LAA are available only for specific commercial models of ultrasound diagnostic machines and are not universal and comparable with other models of machines, pending further development and expansion.

## 5 Conclusion

TrueVue Glass, a new advanced 3D ultrasound imaging technology, provides the first real-time dynamic and radiation-free visualization of the entire external stereoscopic profile of the LAA and adjacent structures with a high degree of consistency with CCTA display and a more complete visualization of the occluder surrounding residual shunts during postoperative follow-up. AI-assisted 3D Auto LAA software streamlines the LAA measurement process, making preoperative LAAO evaluation more efficient and convenient while ensuring accurate and reproducible results. The combination of TrueVue Glass and 3D Auto LAA allows for a more accurate and efficient preoperative assessment of LAA anatomy and physiological parameters for LAAO.

## Data Availability

The original contributions presented in the study are included in the article/Supplementary Material, further inquiries can be directed to the corresponding author.

## Supplementary material

SUPPLEMENTARY MOVIE S1 TrueVue Glass shows morphological changes of left atrium appendage with cardiac cycle. LA, left atrium; LAA, left atrium appendage.

SUPPLEMENTARY MOVIE S2 TrueVue Glass shows the autonomic echogenic reflection in left atrium and left atrium appendage of patient with atrial fibrillation (arrows). LA, left atrium; LAA, left atrium appendage; LV, left ventricle.

## Abbreviations

2D, two-dimensional; 3D, three-dimensional; 3D auto LAA, three-dimensional automated left atrial appendage measurement; AI, artificial intelligence; AF, atrial fibrillation; CCTA, cardiac computed tomographic angiography; LAAO, left atrial appendage occlusion; LZ, landing zone; RT3D, real-time three-dimensional; TEE, transesophageal echocardiography.
